# ReverseGWAS identifies combined phenotypes associated with a genotype in GWA studies

**DOI:** 10.1093/bioinformatics/btag079

**Published:** 2026-02-17

**Authors:** Leonid Chindelevitch, Åsa K Hedman, Dmitri Bichko, Daniel Ziemek

**Affiliations:** MRC Centre for Global Infectious Disease Analysis, School of Public Health, Imperial College, London W2 1NY, United Kingdom; Inflammation and Immunology, Pfizer Research and Development, Cambridge, MA 02139, United States; Inflammation and Immunology, Pfizer Research and Development, Cambridge, MA 02139, United States; Inflammation and Immunology, Pfizer Research and Development, Cambridge, MA 02139, United States

## Abstract

**Motivation:**

Traditional genome-wide association studies (GWAS) aim to uncover the genetic variants associated with a single phenotype of interest (typically a disease), and to elucidate its genotypic architecture. However, many of today’s GWAS simultaneously measure multiple related phenotypes, leading to the possibility of pursuing the reverse aim of elucidating the “phenotypic architecture” of a single genetic variant. In other words, we may ask what combination of measured phenotypes is associated with a given genotypic variant. ReverseGWAS is an algorithmic platform for answering such questions in the context of large-scale multi-phenotype GWAS.

**Results:**

We demonstrate the effectiveness of ReverseGWAS on simulated data, showing its ability to identify logical combinations of phenotypes with a reasonable amount of noise. We then apply it to a selection of combined phenotypes from the UK Biobank, obtaining 719 candidate associations using autoimmune diseases and 205 using common ICD10 codes. We find that the majority of these associations (546/719 and 111/205, respectively) successfully replicate in an independent cohort, FinnGen.

**Availability and implementation:**

The source code of ReverseGWAS is freely available to non-commercial users as an installable R package at https://github.com/Leonardini/rgwas.

## 1 Introduction

Traditionally, a genome-wide association study (GWAS) examines the statistical association of a number of genetic variants with a single phenotype of interest, by collecting data from a cohort of cases (individuals with the phenotype) and controls (individuals without it) (Pearson and Manolio 2008). This approach has been successful in elucidating the genetic underpinnings of some human diseases, such as Hepatitis C (Lu *et al.* 2014) and coronary artery disease (Coronary Artery Disease Genetics Consortium 2011).

In some cases, the association tested may involve multiple genetic variants in combination, with an additive model (where each genetic variant exerts some influence on the phenotype independently of the presence or absence of the other variants) most commonly assumed to govern such a combination. This model can then be turned into a polygenic risk score for the phenotype of interest (Lewis and Vassos 2020).

Given the challenges of recruiting and testing large cohorts, researchers often simultaneously measure multiple phenotypes within the same cohort, allowing multiple GWA studies to be performed at the same time (Burton *et al.* 2007). This trend has culminated in the creation of large-scale biobanks, where subjects are recruited from the general public and simultaneously tested for several hundred phenotypes, with detailed questionnaires probing their demographic characteristics. The most prominent examples include the UK Biobank (Littlejohns *et al.* 2019) and the FinnGen cohort (Locke *et al.* 2019).

Such a multiplicity of phenotypes creates the opportunity to modify the GWAS approach by interrogating combinations of phenotypes for association with specific genetic variants, rather than the traditional approach of interrogating combinations of genetic variants for association with specific phenotypes. While few clear-cut examples of a single genetic variant being associated with multiple phenotypes at the same time are known, such pleiotropic effects may be a common occurrence in some areas, such as psychiatric disorders (Lee *et al.* 2021).

Previous work has examined linear combinations of phenotypes, analogous to the additive model for genetic variants (Ruotsalainen *et al.* 2021). However, linear combinations applied to binary phenotypes may be challenging to interpret. In this work, we propose a novel method, ReverseGWAS, which examines logical combinations of binary phenotypes. These combinations are more interpretable and easier to apply to other cohorts as they contain no coefficients (weights). For example, the meaning of having disease A or disease B is immediately apparent, in a way that having a high value for a weighted average of the two diseases may not be.

We begin by simulating our novel approach, based on integer linear programming (Nemhauser and Wolsey 1988), on a randomization of a subset of UKBB data. We find that our method consistently provides good performance that matches or exceeds that of standard GWAS on combined phenotypes consisting of the simultaneous presence of two pairs of phenotypes, each pair described by or as before. We then apply our method to two distinct sets of phenotypes—immune traits and common ICD10 codes—in UKBB, and replicate the most promising findings in the FinnGen cohort, finding that the majority of combined phenotypes successfully replicate.

We conclude that ReverseGWAS is a promising method to discover new associations involving genetic variants and combinations of phenotypes. As all of our code is publicly available, our results can be independently reproduced by any researcher with access to the UKBB and FinnGen cohorts.

## 2 Materials and methods

The key notation used in this section is displayed in Table 1.

**Table 1:** Summary of key notation used.

Symbol	Meaning	Symbol	Meaning
*K*	n. clauses	*c*	Combined phenotype
*L*	n. items per clause	*i*	Subject index
*M*	n. subjects	*j*	Phenotype index
*N*	n. genotypes	*k*	Clause index
*P*	n. phenotypes	*f*	Association statistic
*X*	Genotype matrix	*u*	A phenotype
*Y*	Phenotype matrix	*v*	A genotype
$S,n_{u}$	n. subjects with combined phenotype		
$T,n_{uv}$	n. subjects with genotype and combined phenotype		
*[R]*	The set {1,…,R} for any positive integer *R*		

### 2.1 Finding combined phenotypes associated with an SNP

As in a standard GWAS, there are *M* subjects, each one genotyped at *N* SNPs, and the information is collected into a matrix $X\in B^{M\times N}$, where B is the set of values. We assume that B is the set $B_{2}:=\{0,1\}$ of binary values, i.e. *X* is an SNP presence/absence matrix, although our methods would also apply with minimal changes to the set $B_{3}:=\{0,1,2\}$ of ternary values, where *X* is an SNP dosage matrix instead. There are also *P* phenotypes for each subject, collected into a matrix $Y\in B^{M\times P}$, also with $B=B_{2}$ (i.e. binary phenotypes).

Formally, the goal of a standard GWAS is to identify, for a particular phenotype $Y_{k}$, the indices *j* such that the corresponding SNP $X_{j}$ is associated with $Y_{k}$, with the strength of association measured by an appropriate statistic; these statistics are discussed in detail in what follows. Sometimes, multiple variables $X_{j_{1}},\ldots ,X_{j_{t}}$ are simultaneously associated with a phenotype, and they can then be linearly combined (i.e. multiplied by coefficients then added) into a ‘polygenic risk score’ (Igo *et al.* 2019, Lewis and Vassos 2020).

In contrast, the goal of ReverseGWAS is to identify, for a particular SNP $X_{j}$, the indices *k* such that the corresponding phenotype $Y_{k}$ is associated with $X_{j}$. If we only seek single genotype-phenotype associations, this is identical to the standard GWAS problem; however, analogously to risk scores, ReverseGWAS seeks combinations of phenotypes that are simultaneously associated with an SNP. Unlike risk scores, however, ReverseGWAS combines the phenotypes logically rather than linearly; this differentiates it from related approaches such as MultiPhen (O’Reilly *et al.* 2012), CLC (Sha *et al.* 2019) or LCP (Ruotsalainen *et al.* 2021).

Additional related work includes PheWAS, a framework for carrying out GWAS-like analysis, but with multiple separate phenotypes, rather than a single combination phenotype, arising from a genotype (Roden 2017) and MANOVA (Multivariate Analysis of Variance) tests, which carry out a multivariable analysis of variance in multiple phenotypes arising from the variance in a single genotype (Stephens 2013). We also note a method of the same name as ours (Dahl *et al.* 2019), whose goal, however, is to identify subtypes of a phenotype using genetic data, rather than to combine several phenotypes.

Other approaches bearing a conceptual similarity to ours include two recent methods, which infer phenotype groups or clusters from networks constructed using both genotype and phenotype information (Xie *et al.* 2023, Cao *et al.* 2024). Importantly, none of these methods considers logical combinations of phenotypes as we do here, making those unique to our approach.

The combined phenotypes we consider here are described by a conjunctive normal form (CNF) (Russell and Norvig 2021). More specifically, we say that a combined phenotype is a (K,L)-CNF if it contains exactly *K* clauses, each containing exactly *L* phenotypes, and the phenotypes within a clause are combined via an or operator while the clauses themselves are combined via an and operator. Although this may seem to be a restriction, it is known (Russell and Norvig 2021) that any (classical) logical expression can be translated into an equivalent CNF.

There are exactly $N_{K,L}:=\left(\begin{array}{c} \left(\begin{array}{c} N\\ L \end{array}\right)\\ K \end{array}\right)$ possible (K,L)-CNF combinations if the phenotypes in a clause and the clauses themselves must be distinct. By allowing repetitions within a clause and repeated clauses, we represent all CNF combinations with ≤K clauses, each one with ≤L phenotypes, meaning the search space for (K,L)-CNF also includes $\left(K^{\prime },L^{\prime }\right)$-CNF whenever $K^{\prime }\leq K$ and $L^{\prime }\leq L$. Consider a hypothetical combined phenotype with K=2 clauses and L=2 phenotypes each, say (A or B) and (C or D). The patients matching the first clause are those with either *A* or *B*, while the patients matching the second clause are those with either *C* or *D*. The patients with the combined phenotype then need to match both; they are those with at least one phenotype among *A* and *B* as well as at least one among *C* and *D*.

### 2.2 A preprocessing step to reduce input complexity

Our inputs are as follows: *X*, a binary M×N genotype matrix; *Y*, a binary M×P phenotype matrix; positive integers *K* and *L*; and the association statistic *f* to optimize. We assume that this statistic f(u,v) for a phenotype *u* and a genotype *v* depends only on the 2×2 contingency table of *u* and *v—*or, equivalently, only on $n_{u}$, the number of 1’s in *u*, $n_{v}$, the number of 1’s in *v*, and $n_{uv}$, the number of 1’s in common between *u* and *v*. Note that $n_{v}$ only depends on the genotype and can be computed independently of phenotype combinations.

A preprocessing step to reduce the running time involves grouping together subjects with identical phenotype patterns (rows of *Y*) and replacing them with a single subject. This is particularly helpful when the number of phenotypes *P* is smaller than ${\;\text{log}\;}_{2}(M)$, since there can be at most $2^{P}$ distinct phenotypic patterns. We therefore collapse identical rows of *Y*, make the corresponding changes to *X*, and explain how the statistics sufficient for evaluating *f* can be derived from this reduced input, in Algorithm 1:

Algorithm 1 Collapse subjects with identical profiles without loss of information. **Ensure:**  X,Y both have *M* rows; *X* has *N* columns

1: π←lexOrder(Y)  ▹ A permutation sorting the rows of *Y*

2: $X^{\prime }\leftarrow \pi (X);Y^{\prime }\leftarrow \pi (Y)$  ▹ Permute the rows of X,Y by π

3: $\left(m,Y^{\prime \prime })\leftarrow \mathrm{runLengthEncode}(Y^{\prime }\right)$  ▹ Encode unique rows

4: $X^{\prime \prime }\leftarrow \mathrm{blockColSums}(X^{\prime },m)$  ▹ Sum the block columns

5: **return**  $\left(X^{\prime \prime },Y^{\prime \prime },m\right)$.

Here, line 3 assumes a subroutine called runLengthEncode operating over the rows of $Y^{\prime }$; it collapses the adjacent identical rows of $Y^{\prime }$ to obtain a reduced representation $Y^{\prime \prime }$ consisting of its unique rows, and also returns the vector *m* with $m_{i}$ being the multiplicity of the *i*th row of $Y^{\prime \prime }$ in $Y^{\prime }$. Line 4 also assumes a subroutine called blockColSums which splits the rows of $X^{\prime }$ into the blocks defined by the multiplicity vector *m*, then replaces each block by its column sums, and finally aggregates these sums as rows into a reduced integer matrix $X^{\prime \prime }$. Note that $X^{\prime \prime }$ and $Y^{\prime \prime }$ have |m| rows, where |m| is the dimension of *m*.

We explain how the reduced inputs can be used in an integer linear programming approach in the next subsection.

### 2.3 An integer linear programming formulation for optimal combined phenotypes

Integer linear programming (ILP) is a widely used paradigm in optimization, specifically in situations where certain variables need to take on integer rather than arbitrary real values (Nemhauser and Wolsey 1988). It is a special case of the constraint satisfaction programming paradigm (Russell and Norvig 2021), which formulates problems as optimization over decision variables satisfying certain constraints. Our problem can be framed as the decision about which phenotypes should be included in each clause, and thus lends itself naturally to this paradigm. We now describe how we convert an instance of our problem for binary genotypes and phenotypes into an ILP, with the objective function to follow.

We state the formulation for a single genotypic variant, *X*. The variables are as follows:

-
$U_{jk},j\in [P],k\in [K]$ is 1 if phenotype *j* is used in clause *k*.
-
$P_{ik},i\in [M],k\in [K]$ is subject *i’*s phenotype for clause *k*.
-
$P_{i},i\in [M]$ is subject *i’*s overall combined phenotype value.
- *S* encodes $n_{u}$, the number of subjects with the phenotype.
- *T* encodes $n_{uv}$, the number of subjects with both the genotypic variant and the phenotype.

There are a total of *KP* variables of the first type, *KM* variables of the second type, *M* of the third type; all of these are binary. The two additional variables *S* and *T* are integers. The constraints enforced on these variables ensure consistency between the variables and their intended meaning, so that the values assigned to the variables define a valid (K,L)-CNF phenotype. These constraints fall into three categories:

The or constraints ensure that each subject *i’*s phenotype is correctly determined for each clause *k* in the combined phenotype. Specifically, if subject *i* has phenotype *j*, and phenotype *j* is present in clause k, the corresponding variable $P_{ik}$ is forced to 1, whereas if none of the phenotypes that subject *i* has is present in clause *k*, then $P_{ik}$ is forced to 0. There are K(M+O) such constraints, where we denote by *O* the total number of 1’s in the phenotype matrix *Y*.

The and constraints ensure that each subject *i’*s phenotype is correctly determined for the overall combined phenotype. Specifically, if $P_{ik}$ is 0 for any clause *k*, meaning that the combination is absent in subject *i*, then $P_{i}$ is forced to 0, whereas if $P_{ik}$ is 1 for every clause *k*, meaning that all of the combinations are present in subject *i*, then $P_{i}$ is forced to 1. There are a total of (K+1)M such constraints.

The constraints on *S* and *T* are count constraints; *S* counts the subjects with the combined phenotype is present, while *T* counts the subjects with the combined phenotype and the genotype of interest. The final constraint ensures that no more than *L* phenotypes are used to define every clause in the combined phenotype. There are K+2 count constraints.

- or constraints; $P_{ik}=1$ iff a phenotype *j* with $Y_{ij}=1$ is in clause *k*:
  $$\begin{array}{c} U_{jk}-P_{ik}\leq 0,\;i\in [M]\;s.t.\;Y_{ij}=1,k\in [K];\\ P_{ik}-\sum_{\left\{i|Y_{ij}=1\right\}}U_{jk}\leq 0\;i\in [M],k\in [K]. \end{array}$$
- and constraints; $P_{i}=1$ iff $P_{ik}=1$ for each clause *k*:
  $$\begin{array}{c} P_{i}-P_{ik}\leq 0,i\in [M],k\in [K];\\ \sum_{k}P_{ik}-P_{i}\leq (K-1),i\in [M]. \end{array}$$
- count constraints; S,T are correctly defined, clause sizes at most *L*:
  $$\begin{array}{c} S-\sum_{i=1}^{M}P_{i}=0;\\ T-\sum_{\left\{i|X_{i}=1\right\}}P_{i}=0;\\ \sum_{j=1}^{P}U_{jk}\leq L\;\forall \;k\in [K]. \end{array}$$

Overall, the formulation contains K(M+P)+M+2 variables and K(2M+O+1)+M inequality constraints plus two equality constraints. However, as described in the next subsection, a few additional variables and constraints may need to be introduced to accommodate a non-linear association statistic.

Since some solutions may include redundant elements in a clause or redundant clauses, we carry out a post-processing step which removes any repeated phenotypes within a clause and only keeps the distinct set-minimal clauses, as any superset clause is implied by a subset clause.

We also collapse subjects with identical phenotype profiles in the ILP formulation using Algorithm 1, and then modify the computation of *S* and *T* to account for multiplicities. Specifically, each $P_{i}$ gets a coefficient equal to the multiplicity of the *i*th subject profile among all subjects (among the subjects with the genotype *X*) in the sum constraints defining *S* (*T*), respectively. This data-driven problem size reduction is implemented as the default approach.

### 2.4 Association statistics in the ILP formulation

Most genotype-phenotype association statistics can be computed from $n_{u},n_{v}$, and $n_{uv}$. We formalize this in Lemma 1, proven in Appendix A, available as supplementary data at *Bioinformatics* online.

Lemma 1. *The following association statistics of a pair of binary vectors u and v of the same size N can be derived from the number of 1’s in u, the number of 1’s in v, and the number of 1’s that u and v have in common: agreement, Hamming distance, covariance, correlation*, $\chi^{2}$  *coefficient, odds ratio, Cohen’s kappa, and Fisher’s exact one-sided P-value.*

Therefore, in terms of the formulation in the previous subsection, the objective function depends only on *S* and *T*. However, an ILP only admits linear objective functions, and the proof of Lemma 1 shows that only the agreement, Hamming distance and covariance are linear in $n_{u}$ and $n_{uv}$, while the other association statistics depend on them non-linearly.

For this reason, instead of directly optimizing an association statistic, we may adopt an indirect approach by ensuring that a bound, such as the Fisher’s exact one-sided *P*-value not exceeding some threshold value $p_{0}$, is satisfied. As we explain in the next subsection, such bounds can be translated into additional constraints in our formulation.

For the rest of this paper, we require the association statistic *f* to be monotonic (either monotone increasing or monotone decreasing) in $n_{uv}$ for fixed $n_{u}$ and $n_{v}$. Monotonicity implies that for fixed $n_{u}$ and $n_{v}$ and bounds *L* and *U*, the set of $n_{uv}$ for which L≤f≤U is an interval. We establish monotonicity for the Fisher’s exact one-sided *P*-value in Lemma 2. It is easily seen from the proof of Lemma 1 that all the other association statistics we consider except the $\chi^{2}$ coefficient are monotonic.

Lemma 2. *The Fisher’s exact one-sided P-value is monotone decreasing in* $n_{uv}$  *for fixed values of* $n_{u}$  *and* $n_{v}$.

### 2.5 Bounds on association statistics in our formulation

To enforce the threshold constraint it is sufficient to ensure that, for each value of *S*, the corresponding value of *T* exceeds a threshold value g(S), also assumed to be integer, where *g* is a deterministic function (essentially a level curve of *f*) that can be easily computed. Our implementation uses a binary search to determine *g* when *f* is the Fisher’s exact one-sided *P*-value.

Consider the collection P of straight-line segments between the points of the form (S,g(S)) for 0≤S≤M (we call P the boundary of *f*). It suffices to ensure that all and only those points that lie on or above P are feasible in the optimization. This constraint can be enforced by adding two variables per linear segment of the boundary of *g* and additional constraints, as stated in Lemma 3 and proven in Appendix A, available as supplementary data at *Bioinformatics* online.

Lemma 3. *Let g be a piecewise linear function with k segments, and let S and T be non-negative variables. Then the constraint* T≥g(S)  *can be enforced by adding 2k new variables and* k+3  *linear constraints.*

This result suggests that minimizing the number of segments needed to represent the boundary of *f* can help reduce the complexity of the formulation, as the default option can add 2*M* new variables. We achieve this minimization in two stages:

1. Prune the boundary P to make it non-redundant by selecting the subset R consisting of the first (leftmost) segment endpoint along with the endpoints having the maximum value of *S* for each feasible g(S). It is easily seen that $x\in N^{2}$ lies below P if and only if it lies below R.
2. Pick δ>0 and run the Imai-Iri algorithm (Imai and Iri 1986) with width w=1−δ to find the most compact (having the fewest segments) piecewise linear function $\hat{g}$ on or above the points in R and exceeding them by at most *w*. Then $\hat{g}$ is a boundary between the integer points lying strictly below R and those lying on or above R.

Note that the value of δ provides a trade-off between the number of segments in $\hat{g}$ and the numerical stability of the resulting calculations. We empirically set δ=0.001 as a default.

### 2.6 Iterative refinement to optimize non-linear statistics

The previous subsection demonstrates that, for a non-linear monotonic association statistic *f* between a genotype *G* and a combined phenotype *P*, it is possible to ensure that $f_{\min}\leq f(G,P)\leq f_{\max}$ (where either $f_{\min}=-\infty$ or $f_{\max}=\infty$ is allowed) with a small increase in the formulation’s complexity. However, if we wish to optimize f(G,P) over all (K,L)−CNF combined phenotypes *P*, we can use a two-stage strategy:

1. Start by determining whether a *P* with f(G,P) satisfying a crude target bound is feasible. As it can be applied to multiple genotypes of interest, we call this the triage stage.
2. Once it is known that such a *P* exists, check the feasibility of progressively smaller (or larger) values of *f*, first using a doubling method, and finishing with a binary search. Since only feasibility is of interest, any objective function suffices; we have found that the agreement objective performs well in practice. We call this the optimization stage.

The complete algorithmic details are provided in Appendix B, available as supplementary data at *Bioinformatics* online.

### 2.7 Simulations help identify optimal method parameters

Before applying ReverseGWAS to real data, we calibrate it on data that are similar to, but distinct from, the real data. To do so we create a collection of simulated inputs, each containing a randomly constructed (K,L)-CNF phenotype for specified values of *K*, *L* and subject number *M*, with a specified fraction ϵ of its entries logically negated to simulate noise.

Thus, these simulation experiments consider adversarial noise, whereby the presence of an SNP is replaced by its absence or vice versa. They are designed to identify the settings where the method is able to correctly identify the right combined phenotypes while keeping the number of false positives small, and to examine the scaling behaviour of the algorithm as a function of both the sample size and phenotype complexity (measured by *K* and *L*).

Table 2 specifies the set of values for each parameter that we test in the simulation. In total, there are 81 parameter combinations, each one tested in 10 independent trials. The exact process we follow for generating the simulation data while preserving the anonymity of the input genotypic and phenotypic data is described in detail in Appendix C, available as supplementary data at *Bioinformatics* online.

**Table 2:** List of parameters defining the 81 simulation settings.

Parameter	*K*	*L*	*M*	ϵ
Meaning	# Clauses	Clause size	# Subjects	Noise level
Low	1	1	2000	0
Medium	2	2	20 000	0.1
High	3	3	200 000	0.2

We assume that the majority of the 300 000 or so SNPs in the real data do not have a good match among our set of phenotypes. For this reason, the full input to each simulation consists of 50 SNPs, one of which is the pseudo-SNP representing the noisy combined phenotype, and the rest are real randomized SNPs from chromosome 1 (a total of just over 22 000) with a similar minor allele frequency. We deem the application of ReverseGWAS to a particular simulation dataset successful if it is able to identify the pseudo-SNP in the line-up of 50 SNPs.

We apply ReverseGWAS to the simulation data in the same way that we later apply it to real data, namely, by splitting the simulated data into two equal halves referred to as the discovery and validation cohorts. We use it in optimization mode on the discovery cohort to identify promising phenotype combinations, and then test them on the validation cohort.

As a comparison baseline, we also run the standard GWAS methodology (with no adjustment for population structure) on the same input data, and use the same success criteria as for ReverseGWAS. Note that because a standard GWAS can only consider a single phenotype at a time, we cannot fairly compare the two methods on whether the combined phenotype they discover is the correct combination of phenotypes.

### 2.8 Real data: exploration and independent replication

We then apply ReverseGWAS to real phenotypic and genetic data to test genetic associations with logical combinations of (i) the 15 most common ICD10 codes intersecting with at least one other code, (ii) a larger set of the 42 most common ICD10 codes, and (iii) 11 autoimmune diseases. The list of phenotypes is provided in Table 1, available as supplementary data at *Bioinformatics* online. Hypertension, the most common ICD10 code, was excluded due to its considerable intersection with many other phenotypes.

We conducted the analysis in two phases: an exploratory phase using UKBB (Sudlow *et al.* 2015) and a replication phase using FinnGen (Locke *et al.* 2019).

In the exploratory phase, we used the UKBB data for around 500 000 people aged between 40 and 69 years, recruited between 2006 and 2010 across the UK. A total of 341 485 of them matched our inclusion criteria of having European ancestry and being mutually unrelated. Individuals were first split into equal-sized discovery and validation sets, and genome-wide significant associations ($P<5\times 10^{-8}$) in the discovery set brought forward for validation.

We focused the genetic association analyses on the 302 349 autosomal SNPs from the Illumina HapMap300 panel remaining after removing the variants fitting any exclusion criteria: missingness rate above 10%, minor allele frequency below 1%, or Hardy–Weinberg equilibrium *P*-value below $10^{-15}$. Samples with a missingness rate above 10% were excluded. The negated version of each SNP was considered in the association analysis for a role similar to that of protective SNPs in standard GWAS.

In the replication phase, putative associations that reached genome-wide significance in both the discovery and validation cohorts in UKBB were analysed using conventional GWAS methods in FinnGen, a large-scale research project that contains genomics and matching health data on 500 000 Finnish individuals (Kurki *et al.* 2023). We recreated the combined phenotypes using the CNF formulas obtained in the exploratory phase, and performed an additive GWAS using Regenie with covariates for age, sex, 10 genetic principal components, array, and batch (Mbatchou *et al.* 2021). Data from FinnGen release 9 were used in the analyses. All the SNP positions we report are based on build 38 of the human genome (hg38).

## 3 Results

### 3.1 ReverseGWAS needs modest computational resources

The time required to execute ReverseGWAS computations was reasonable and comparable to the computation time of standard GWAS via logistic regression, as implemented in GWASTools (Gogarten *et al.* 2012), for the same inputs. In particular, none of the runs with 11 phenotypes and 50 SNPs needed more than 10 minutes in total. A summary of the timings by K (number of clauses), L (number of items per clause), M (number of patients) and eps (noise level) is provided as Table 4, available as supplementary data at *Bioinformatics* online.

The same observation applied to memory consumption; the CPLEX branch-and-bound trees were limited to 20 GB in all of our runs, and none of them has ever returned an out-of-memory error code, meaning this amount was always adequate.

### 3.2 ReverseGWAS can outperform GWAS in simulation

As per Table 2, there are 81 parameter combinations, and each one is processed against 10 randomizations of the phenotype matrix. Each simulated dataset contains 50 SNPs, so we use a Bonferroni-corrected significance threshold, P<.05/50, for the one-sided Fisher’s exact test. For each SNP that attains this threshold for some combined phenotype with the parameters *K* and *L* used to generate it, we then optimize the *P*-value to get the most highly associated combined phenotype, and assume that it is the correct one. The pseudo-SNP corresponding to the noisy combined phenotype is a true positive if it reaches this threshold, and a false negative otherwise; any other SNP reaching this threshold is a false positive.

We then look at all the SNP-combined phenotype pairs that reach the same significance threshold, P<.05/50, on the validation cohort. The validation cohort serves two related purposes. First, it allows us to determine which combined phenotypes happened to have a strong association with the input SNP in the discovery cohort by chance, therefore helping to control overfitting. Second, it allows us to compare the ReverseGWAS method with the standard GWAS by looking at the strongest single-phenotype association for each SNP in the discovery cohort, and assessing it in the validation cohort, helping us to determine the value added by using ReverseGWAS to discover the combined phenotypes.

Overall, across the 810 simulations, 3743 SNPs out of a total of 39 690 spurious SNPs (9%) were false positives in the discovery cohort, and of those, only two reached the same threshold in the validation cohort, both in the K=L=3 setting, the most likely one to lead to overfitting. This finding suggests that ReverseGWAS controls false positives well despite its larger number of degrees of freedom compared to GWAS.

On the other hand, 151 out of 810 pseudo-SNPs (19%) were false negatives as they could not reach a *P*-value of .05/50 in the discovery cohort, with the false-negative rate increasing with noise (42 with ϵ=0, 50 with ϵ=0.1, and 59 with ϵ=0.2) and decreasing with the number of subjects (107 with M=2000, 32 with M=20 000, and 12 with M=200 000). False-negative rates decreased (increased) slightly with *K* (*L*).

Among the pseudo-SNPs that were successfully identified in discovery, 568 out of 659 (86%) also reached the target *P*-value threshold in the validation cohort. Among the 91 that did not reach it in the validation stage, there was an even stronger trend for association with noise level (14 with ϵ=0, 31 with ϵ=0.1 and 46 with ϵ=0.2), as well as with the number of phenotypes per clause *L* (1 with L=1, 29 with L=2 and 61 with L=3), but no clear trend for association with the number of clauses *K* or the number of subjects *M*. The overall two-stage true-positive rate of ReverseGWAS was 568 out of 810, or over 70%.

For comparison, the standard GWAS approach identified a lot fewer spurious SNPs in the discovery cohort (197/39 690, or 0.5%) due to its more restricted nature; only four of these reached the significance threshold in the validation cohort. Standard GWAS also identified fewer true positives: 528 out of 810 (62%) reached significance in the discovery stage, and 472 of them (89%) reached it in the validation stage as well. The overall true-positive rate for standard GWAS was 472 out of 810, or 58%. In addition, 308 out of 472 (65%) of the validated associations were found in the simpler settings with either K=1 or L=1.

**Figure 1:**
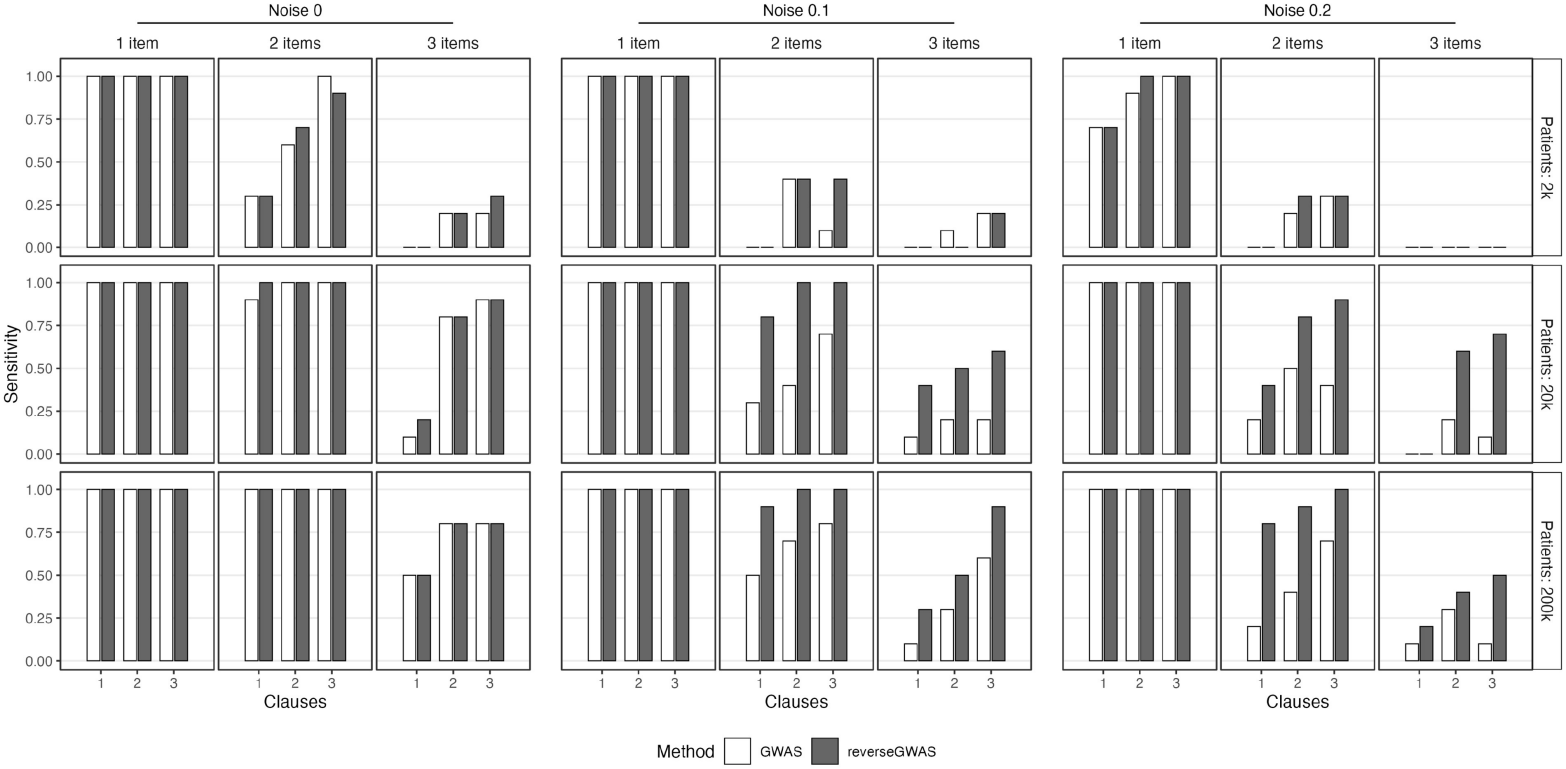
True-positive rate (sensitivity) for the GWAS (white) and ReverseGWAS (black) methods. For each number of items (*L*), the results are arranged by number of clauses (*K*). The results are faceted by the number of subjects *M* (rows) and the noise level ϵ (columns).

The overall true-positive rates for the two approaches are shown in Fig. 1; the true negative rates are not shown because they are nearly 100% across all simulations. When comparing across parameter combinations, we observed the best signal amplification relative to standard GWAS in the K=2,L=2 setting when aggregated across noise levels and subject numbers, with a gain of almost 40% in true-positive rate (from 51/81 to 71/81). For this reason we decided to proceed with the K=2,L=2 setting when working on the real data.

We have also carried out an additional simulation in which gene dosage information was made available to the GWAS method, but not to ReverseGWAS, which only considered binary genotype (i.e. SNP presence/absence via dominant coding). This alternative simulation, described in more detail in Appendix C, available as supplementary data at *Bioinformatics* online, showed that the additional information available to GWAS did not overcome its main limitation relative to ReverseGWAS, that of considering one phenotype at a time. The results, shown in Fig. 1, available as supplementary data at *Bioinformatics* online, were qualitatively similar to those reported here.

### 3.3 Most UKBB associations from ReverseGWAS replicate

We applied ReverseGWAS to 302 349 SNPs genome-wide in up to 341 485 unrelated European ancestry individuals in the UKBB (Sudlow *et al.* 2015), focusing on combined phenotypes of prevalent ICD10 codes (341 485 individuals) or autoimmune diseases (281 591 individuals). These phenotypes are listed in Table 1, available as supplementary data at *Bioinformatics* online. Associations between combined phenotypes and SNPs found to be significant in the discovery cohort and in the validation cohort in UKBB (a total of 582 independent combined phenotype—SNP associations at the genome-wide significance threshold $P<5\times 10^{-8}$) were then externally replicated in the FinnGen cohort (Kurki *et al.* 2023).

Overall, we were able to reconstruct 551 of the 582 combinations (95%) based on the genotypic variants available in FinnGen, and 518 (94%) of them replicated at an appropriate significance threshold. We now describe some of those replicated phenotypes in detail.

### 3.4 Few ICD10 code associations, but most also replicate

Applying ReverseGWAS to the most common ICD10 codes in the UKBB (excluding hypertension) with a genome-wide significance threshold of $p<5\times 10^{-8}$ resulted in 46 associations involving 46 distinct combined phenotypes composed of 37 unique ICD10 codes, and 32 distinct SNPs, 19 of them negated. Table 2, available as supplementary data at *Bioinformatics* online contains the complete list.

**Figure 2:**
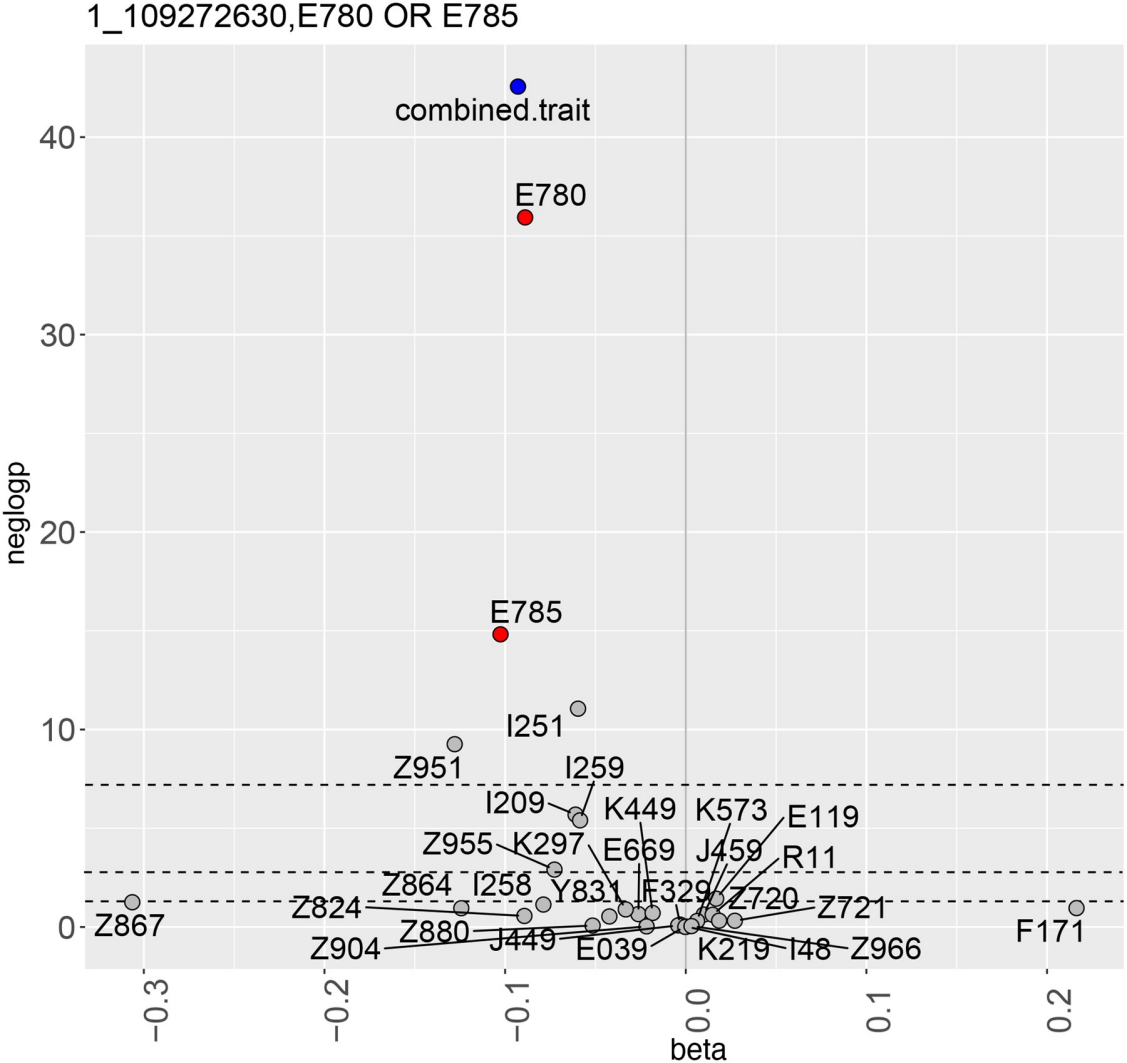
Volcano plot of an association in FinnGen near the CELSR2 locus with a combined phenotype of (E780 or E785), i.e. hypercholesterolaemia or hyperlipidaemia, unspecified. Statistics shown for the combined phenotype (blue), its component phenotypes (red), and the remaining phenotypes (grey).

In external replication using conventional GWAS methods, 34 of 46 associations could be tested. Of these, 32 replicated at a Bonferroni-adjusted significance threshold, p=0.05/46, with the same direction of effect (see Table 2, available as supplementary data at *Bioinformatics* online). Replicating signals included associations of E780 (pure hypercholesterolaemia) or E785 (hyperlipidaemia, unspecified) with rs611917, an SNP nearest to the CELSR2 gene previously reported as a susceptibility locus for cholesterol (Klarin *et al.* 2018). Figure 2 shows the synergy of the combined phenotype relative to the individual phenotypes via association statistics.

### 3.5 Most autoimmune trait SNPs are in the HLA region

Applying ReverseGWAS to 11 autoimmune diseases with a genome-wide significance threshold of $P<5\times 10^{-8}$ resulted in 536 associations involving 129 distinct combined phenotypes and 469 distinct SNPs. The complete list appears in Table 3, available as supplementary data at *Bioinformatics* online. The majority of these (467 out of 536 associations, or 87%) were in the HLA (Human Leukocyte Antigens) region. In external replication, 517 out of 536 combinations could be tested. Out of these, 486 (94%) replicated at a Bonferroni-adjusted threshold (P=.05/536) with the same direction of effect. The remaining combined phenotype—SNP combinations were either too rare or the exact SNP was missing in the FinnGen cohort data.

**Figure 3:**
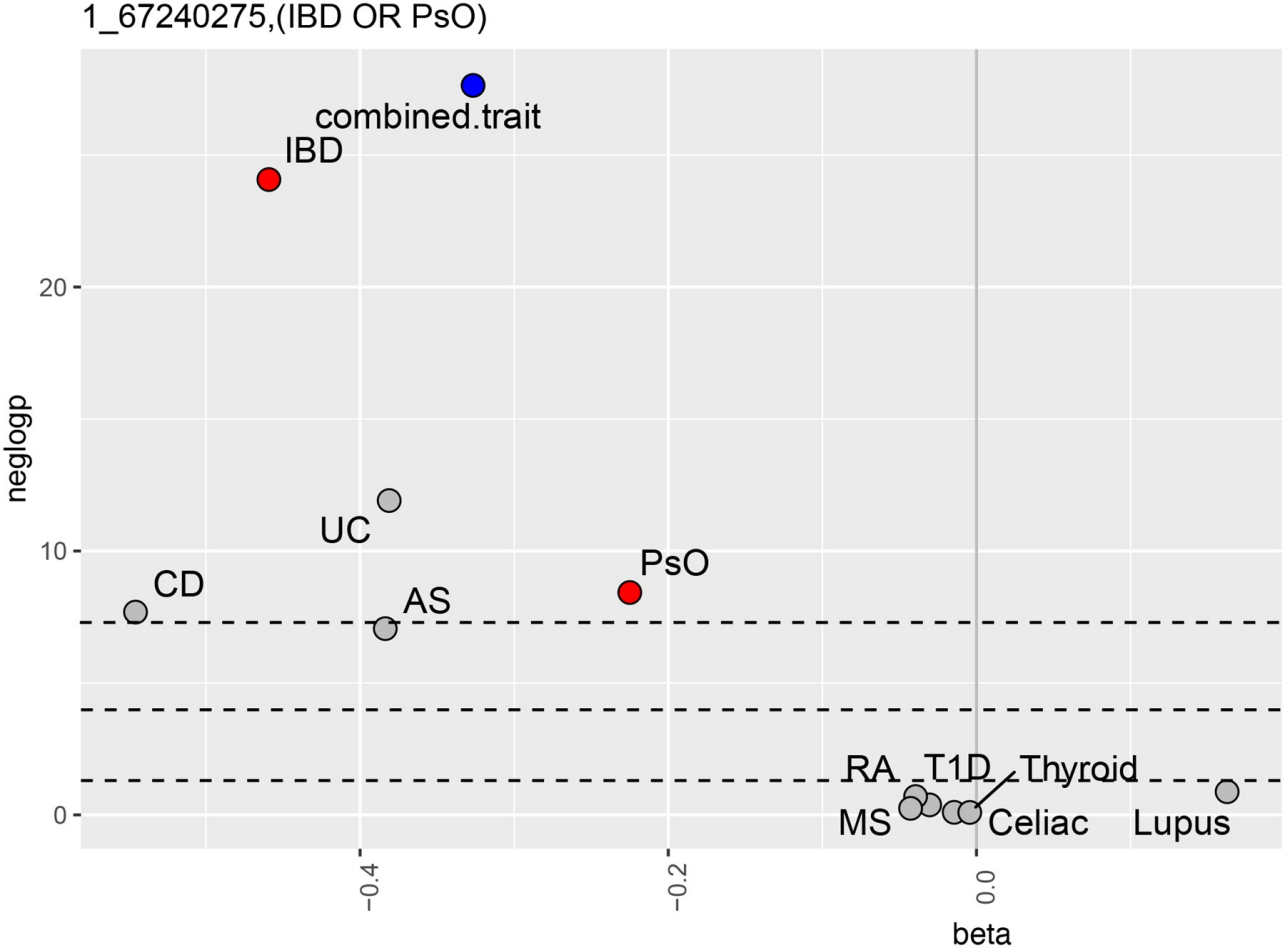
Volcano plot of an association at the IL23R locus (rs11209026) in FinnGen with a combined phenotype (IBD or PsO) and individual autoimmune phenotypes. Statistics shown for the combined phenotype (blue), its component phenotypes (red), and the remaining phenotypes (grey).

Replicating signals outside of HLA included associations close to or in IL23R (Interleukin 23 receptor) and IL2RA (Interleukin-2 receptor alpha chain). Multiple associations were observed for SNPs close to IL23R (Table 3, available as supplementary data at *Bioinformatics* online). The combined phenotype (IBD or PsO) (psoriasis) had associations with two independent SNPs, rs11209026 and rs10889677. This combination is illustrated in Fig. 3.

**Figure 4:**
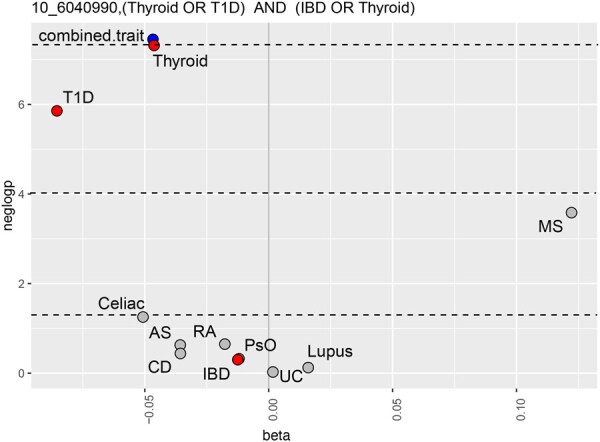
Volcano plot of an association at the IL2RA locus (rs6602398) in FinnGen with combined phenotype (thyroid or T1D) and (IBD or thyroid) and individual autoimmune phenotypes. Statistics shown for the combined phenotype (blue), its component phenotypes (red), and the remaining phenotypes (grey).

A similar combination (IBD or AS) (ankylosing spondylitis), was found to be associated with rs1343151 close to the same gene (results not shown). Genetic variants in this region have previously been associated with susceptibility to PsO (Stuart *et al.* 2022), IBD (Duerr *et al.* 2006), and AS (Reveille et al. 2010). Furthermore, the combined phenotype of (thyroid or T1D) and (IBD or thyroid) was found to be significantly associated with rs6602398 in IL2RA (Fig. 4). Note that the combined trait reaches genome-wide significance while none of its individual components does, even if the margin between its *P*-value and that of thyroid is admittedly small.

Figures 2–4 show statistics (β on the *x*-axis and $-{\;\text{log}\;}_{10}P$ on the *y*-axis) for the combined phenotype (blue), the autoimmune phenotypes or ICD10 codes which are its components (red), and the remaining autoimmune phenotypes or ICD10 codes (grey). Dashed lines indicate significance thresholds: genome-wide (top), Bonferroni-adjusted (middle), and nominal (bottom).

## 4 Discussion

The ReverseGWAS method we propose here innovates on the traditional GWAS paradigm by enabling the search for combined phenotypes correlated with a given genotypic variant, in a principled and scalable way. Unlike previous related work, the strength of ReverseGWAS is the use of logically combined phenotypes, providing a more transparent interpretation and paving the way to novel biological hypotheses. We showed that ReverseGWAS scales to large datasets such as the UK Biobank and identifies combinations that almost invariably replicate in FinnGen, an independent cohort.

Our combined use of data compression (Algorithm 1), a novel reformulation approach (Lemma 3), and the power of the CPLEX solver for 0–1 ILPs has allowed us to apply ReverseGWAS to hundreds of thousands of subjects, genome-wide SNP coverage, and over 40 phenotypes (common ICD10 codes). We expect it to scale to even larger inputs, although it may become slow with many additional phenotypes due to the number of possible combinations.

ReverseGWAS recapitulates known associations both in and out of the HLA region. In particular, SNPs close to IL23R are found to be associated with combinations of the IBD, AS and PsO phenotypes, all of which have been associated with variants in this region (Duerr *et al.* 2006, Reveille *et al*. 2010, Stuart *et al.* 2022). The IL23 receptor in complex with IL12RB1 binds the pro-inflammatory cytokine IL23 primarily on T-helper cells. IL23 plays an important role in inflammatory diseases and is a target of IBD drugs (Tang *et al.* 2012, Verstockt *et al.* 2023).

Our analysis additionally found a genetic variant in IL2RA to be associated with (thyroid or T1D) and (IBD or thyroid). Genetic studies have previously shown this region to be involved in the risk of T1D (Chiou *et al.* 2021), Crohn’s disease (Franke *et al.* 2010), and thyroid disease (Saevarsdottir *et al.* 2020). IL2/IL2RA are known to be important for the function of regulatory T cells and play a critical role in autoimmune diseases (Garg *et al.* 2012).

In total, 38 out of the 42 distinct ICD10 codes appeared at least once among the replicating combinations; however, the majority of these codes (24/38) appeared at most 3 times. The top five most frequently occurring ICD10 codes were Hypothyroidism, unspecified; Atrial fibrillation and flutter; Pure hypercholesterolaemia; T2D without complications; and Asthma, unspecified.

A limitation of our current approach is its focus on the presence/absence data for SNPs, eliminating the distinction between homozygous and heterozygous individuals. It may be interesting to consider gene dosage data in the future; this would require association statistics for 2×3 contingency tables or a logistic regression approach, as commonly used in GWAS (Gogarten *et al.* 2012). However, as our additional simulations show, even dominant coding of dosage data allows ReverseGWAS to outperform the standard method when the correct phenotypes are combination phenotypes.

A further limitation is that the discrete nature of the phenotypes expected by ReverseGWAS currently precludes its use on quantitative traits, such as height or the concentration of a metabolite. However, if those traits can be meaningfully binarized (e.g. tall/short individuals or high/low metabolite concentration), ReverseGWAS can still be useful for identifying promising combinations of those traits given a set of SNPs.

While our simulation results suggest a benefit to ReverseGWAS beyond standard GWAS, and our replication results corroborate this, some signals may still be driven by single associations. For this reason, we suggest exploring the use of the Cochran-Mantel-Haenszel (CMH) test, in effect treating the single best (most associated) phenotype as a confounder (Vierra *et al.* 2023) in the genotype to combined phenotype association. Lower CMH test *P*-values may indicate that this association is unlikely to be driven by the single best phenotype. These *P*-values are output by ReverseGWAS by default, and their use for prioritizing combinations is a promising direction for future research.

In summary, ReverseGWAS provides a novel paradigm for the efficient, scalable, and robust discovery of interpretable associations between combined phenotypes and genotypic variants in large multi-phenotype GWA studies, thus expanding the space of possible associations beyond the single-phenotype model and the harder to interpret linear combinations. We generally recommend setting (K,L)=(2,2) to avoid overfitting, but (K,L)=(3,1) and (K,L)=(1,3) are also worth exploring. The packaged R code is freely available to non-commercial users at https://github.com/Leonardini/rgwas.

## Supplementary Material

btag079_Supplementary_Data

## Data Availability

The data underlying this article are available via UK Biobank and the FinnGen Consortium, and can be accessed on application to the relevant governing body. All code, including the code required to generate the simulation data, is available at https://github.com/Leonardini/rgwas.
