# Supplementary material for: ReverseGWAS identifies combined phenotypes associated with a genotype in GWA studies
Source: Bioinformatics. 2026 Feb 17;42(3):btag079. doi: 10.1093/bioinformatics/btag079 (PMC13003317; doi:10.1093/bioinformatics/btag079)
Supplement: btag079_Supplementary_Data [file btag079_supplementary_data.zip › ReverseGWASAppendixOnly.pdf]

## Appendix A: Proof of the lemmas

**Lemma 1** The following association statistics of a pair of binary vectors $u$ and $v$ of the same size $N$ can be derived from the number of 1's in $u$, the number of 1's in $v$, and the number of 1's $u$ and $v$ have in common: agreement, Hamming distance, covariance, correlation, $\chi^2$ coefficient, odds ratio, Cohen's kappa, and Fisher exact one-sided $p$-value.

*Proof* To begin with, the knowledge of $N$, $n_u$, $n_v$ and $n_{uv}$ allows us to compute the full $2 \times 2$ contingency table between $u$ and $v$:

$$T[u,v] := \begin{pmatrix} n_{uv} & n_u - n_{uv} \\ n_v - n_{uv} & N - n_u - n_v + n_{uv} \end{pmatrix}.$$

The agreement $A(u,v)$ scores $+1$ for the positions where $u$ and $v$ agree and a $-1$ for the positions where they disagree, and can be expressed as

$$A(u,v) = N + 4n_{uv} - 2n_u - 2n_v.$$

The Hamming distance can be derived from the agreement via

$$d_H(u,v) = \frac{N - A(u,v)}{2}.$$

The covariance between $u$ and $v$ is given by

$$Cov(u,v) := \frac{1}{N} n_{uv} - \frac{1}{N^2} n_u n_v.$$

The correlation between $u$ and $v$ is given by

$$\rho(u,v) := \frac{N^2 Cov(u,v)}{\sqrt{n_u(N - n_u)}\sqrt{n_v(N - n_v)}}.$$

The $\chi^2$ statistic is given by

$$\chi^2(u,v) := N\rho^2(u,v).$$

The odds ratio between $u$ and $v$ is given by

$$OR(u,v) := 1 + \frac{NCov(u,v)}{(n_u - n_{uv})(n_v - n_{uv})}.$$

Cohen's kappa for the agreement between $u$ and $v$ is

$$\kappa(u,v) := \frac{2NCov(u,v)}{N(n_u + n_v) - 2n_u n_v}.$$

Lastly, although there is no closed form for the Fisher exact one-sided $p$-value, it can be computed directly from the table $T[u,v]$. □

**Lemma 2** The Fisher exact one-sided $p$-value is monotone decreasing in $n_{uv}$ for fixed values of $n_u$ and $n_v$.

*Proof* As the proof of Lemma 1 states, the Fisher exact one-sided $p$-value can be computed directly from the table $T[u,v]$. This $p$-value is obtained by adding the probabilities of all $2 \times 2$ tables with the same row sums, $n_u$ and $N - n_u$, and the same column sums, $n_v$ and $N - n_v$, whose top right entry is greater than or equal to $n_{uv}$. Recall that $n_u$ and $n_v$ are fixed by hypothesis. Since the probability of each table is non-negative, and increasing $n_{uv}$ eliminates some terms in the sum defining the one-sided $p$-value, it follows that this $p$-value is indeed monotone decreasing.

To restate this slightly differently, the one-sided $p$-value is the right tail sum of a hypergeometric distribution whose parameters are fixed once we fix $n_u$ and $n_v$, and thus increasing the left boundary of this sum shifts the tail to the right and can only decrease the sum defining this $p$-value. □

**Lemma 3** Let $g$ be a piecewise linear function with $k$ segments and let $S$ and $T$ be non-negative variables. Then the constraint $T \geq g(S)$ can be enforced by adding $2k$ new variables and $k+3$ linear constraints.

*Proof* We follow the note on SOS2 constraints in GLPK (Makhorin, 2008).

We introduce binary variables $z_t$ for each $1 \leq t \leq k$, which indicate the active segment of the piecewise linear function $g$. To ensure that exactly one segment is active we add the constraint

$$\sum_{t=1}^{k} z_t = 1.$$

We introduce the non-negative fraction variables $s_t$ for each $1 \leq t \leq k$, which indicate how far along segment $t$ to go. We add the $k$ constraints

$$s_t \leq z_t, 1 \leq t \leq k,$$

to ensure that this fraction is only non-zero for the active segment.

Assume that segment $t$ of the function $g(\cdot)$ is the straight line from $(x_{t-1}, y_{t-1})$ to $(x_t, y_t)$, for each $1 \leq t \leq k$, with $x_{t-1} < x_t$ for $1 \leq t \leq k$. The next constraint forces $S$ to use up exactly the right fraction $s_t$ of segment $t$ along the $x$-axis:

$$S = \sum_{t=1}^{k} x_{t-1} z_t + (x_t - x_{t-1}) s_t.$$

The final constraint ensures the value of $T$ exceeds $g(S)$ for the segment in which $S$ lies; it is similar, with $y$ replacing $x$:

$$T \geq \sum_{t=1}^{k} y_{t-1} z_t + (y_t - y_{t-1}) s_t.$$

Indeed, setting $z_t = 1$ for some $t$ forces all other $z$ variables to be 0 by the first constraint, and all the $s$ variables to be 0 by the second constraint family, leaving $s_t \in [0,1]$. The constraint on $S$ then simplifies to $S = x_{t-1} + (x_t - x_{t-1})s_t$, while the one on $T$ simplifies to $T \geq y_{t-1} + (y_t - y_{t-1})s_t$. Therefore, if $s_t > 0$, since $x_t > x_{t-1}$,

$$T - y_{t-1} \geq \frac{y_t - y_{t-1}}{x_t - x_{t-1}}(S - x_{t-1}),$$

where the right-hand ratio is the slope of segment $t$, so that $T \geq g(S)$ inside it, while if $s_t = 0$, then $T \geq g(S)$ at the left edge of segment $t$, as required. □

## Appendix B: Details of our optimization method

We use Algorithm 2 for a complete optimization of a non-linear statistic over the full set of SNPs. For simplicity, we consider minimization with an upper bound $f_{\max}$, and use the subroutine **feasSolution**$(f, x, Y, b)$ that returns a logical combination $y$ of the columns of $Y$ with $f(x, y) \leq b$, or $NULL$ if no such combination exists.

**Algorithm 2** Calculate for each $i$ a logical combination $best_i$ over $Y$ minimizing $f(X_i, best_i)$ if the minimum is at most $f_{\max}$.

**Require:** $f$ a monotone statistic of two vector arguments
**Ensure:** $X, Y$ both have $M$ rows; $X$ has $N$ columns
1: $k \leftarrow 0$, $I_0 \leftarrow \{1, \ldots, N\}$, $b_0 \leftarrow f_{\max}$
2: **for** $i \in I_0$ **do**
3: $\quad best_i \leftarrow NULL$
4: **end for**
5: **while** $I_k \neq \emptyset$ **do**
6: $\quad I_{k+1} \leftarrow \emptyset$
7: $\quad$ **for** $i \in I_k$ **do**
8: $\quad\quad Y_i \leftarrow$ **feasSolution**$(f, X_i, Y, b_k)$
9: $\quad\quad$ **if** $Y_i \neq NULL$ **then**
10: $\quad\quad\quad best_i \leftarrow Y_i$
11: $\quad\quad\quad I_{k+1} \leftarrow I_{k+1} \cup \{i\}$
12: $\quad\quad$ **end if**
13: $\quad\quad b_{k+1} \leftarrow b_k/2$, $k \leftarrow k + 1$
14: $\quad$ **end for**
15: **end while**
16: **for** $0 \leq j \leq k - 1$ **do**
17: $\quad$ **for** $i \in I_j - I_{j+1}$ **do**
18: $\quad\quad best_i \leftarrow$ **binSearch**$(b_{j+1}, b_j,$ **feasSolution**$, f, X_i, Y)$
19: $\quad$ **end for**
20: **end for**

Here, the loop in Lines 16 to 20 assumes a generic binary search function **binSearch** whose first three arguments are a lower bound, an upper bound, and a function to be optimised, and whose remaining arguments are passed on to the function (with the missing value being searched on). This loop can be avoided if we only wish to obtain the optimal value of $f$ to within a factor of 2, which is our typical approach when minimizing $p$-values because getting an exact minimal $p$-value runs the risk of overfitting.

## Appendix C: Details of the simulation

We applied randomization to the data in the UK BioBank. This allowed us to anonymize the underlying genotypic and phenotypic data while preserving its general characteristics.

First, we extracted the matrix $Y$ containing the 11 autoimmune phenotypes and created $R = 10$ randomized versions. Each randomized version $Y_r$, with $1 \leq r \leq R$ had the same row and column sums as $Y$, meaning that it contained the same number of phenotypes per subject and the same number of subjects per phenotype as $Y$. The randomization was performed using the Swapping (Switching) Algorithm known since the late 1950s (Ryser, 1957; Besag and Clifford, 1989), and implemented in the BiRewire package in Bioconductor (Gobbi *et al.*, 2022).

Given that each of the phenotypes is present in only a small fraction of the subjects (from 517 for lupus to 17,147 for thyroid, out of 281,591 subjects), we based the selection towards the 37,112 subjects with at least one phenotype by allocating to every subject a probability proportional to 1 + the number of phenotypes they had. We then drew two disjoint sets of size $M$: $D_M$ for discovery subjects and $V_M$ for validation ($2M$ in total) without replacement according to this probability distribution. This was done independently at random for each of the population sizes $M$. Note that due to the randomization defining $Y_r$ the prevalence of each phenotype among the subjects in $D_M$ and $V_M$ may vary with $r$.

After subject selection we created the combined phenotypes by sampling, independently at random for each $(K, L)$ pair and for each randomization $r$, a set of $KL$ phenotypes (with replacement) that we turned into a $K \times L$ matrix. This matrix represented the combined phenotype $x_{K,L,r}$ in CNF, with each row a clause over phenotypes aggregated via an **or**, and the clauses (rows) themselves aggregated via an **and**.

To model the addition of noise we replaced the noiseless combined phenotype $x_{K,L,r}$ with a pseudo-SNP $x_{K,L,r,\epsilon}$. This was done by selecting $\epsilon M$ components of $x_{K,L,r}$ independently at random without replacement, and flipping them so that 1's became 0's and 0's became 1's. This noise could change the minor allele frequency of the combined phenotype by up to $\epsilon$ in either direction. In order to prevent excessive fluctuations due to the addition of noise, we added the same expected amount of noise to both the discovery and the validation cohorts.

We divided the minor allele frequencies (MAFs) into $n = 20$ bins of width $w = 0.05$ and randomly selected, from the just over 22,000 actual SNPs in chromosome 1 in the data, a subset with MAFs in the same bin as the pseudo-SNP $x_{K,L,r,\epsilon}$ in the discovery set $D_M$. For each simulation we used $U = 49$ such SNPs, selected uniformly at random from this bin to form the input matrix $X'$, then randomized it using the Swapping algorithm while preserving the row and column sums to obtain a matrix $X''$. The final input to the simulation was formed by prepending the pseudo-SNP $x_{K,L,r,\epsilon}$ to $X''$ on the genotype side, and by the randomized matrix $Y_r$ restricted to $D_M$ (discovery) and $V_M$ (validation) on the phenotype side.

For the additional simulation, we generated all the data in exactly the same way, but transformed the input data to gene dosage data by randomly assigning a proportion $\pi$ of the SNP values to 2 instead of 1. The proportion $\pi$ was determined so as to keep the SNP in approximate Hardy-Weinberg equilibrium. The GWAS method, as implemented in GWASTools (Gogarten *et al.*, 2012), was provided this dosage data as input, while `ReverseGWAS` treated it as binary. A limitation was that some logistic regression runs (especially noiseless ones with small sample sizes) did not converge, so the GWAS method was evaluated based only on the $p$-values among the runs that did.

## Appendix D: Full acknowledgment statement

We acknowledge the participants and investigators of the FinnGen study (Kurki *et al.*, 2023), funded by two grants from Business Finland (HUS 4685/31/2016, UH 4386/31/2016) and industry partners AbbVie Inc., AstraZeneca UK Ltd, Biogen MA Inc., Bristol Myers Squibb (and Celgene Corporation & Celgene International II Sàrl), Genentech Inc., Merck Sharp & Dohme LCC, Pfizer Inc., GlaxoSmithKline Intellectual Property Development Ltd., Sanofi US Services Inc., Maze Therapeutics Inc., Janssen Biotech Inc, Novartis AG, and Boehringer Ingelheim International GmbH.

We acknowledge the following biobanks, members of BBMRI.fi infrastructure, for delivering samples to FinnGen: Auria Biobank, THL Biobank, Helsinki Biobank, Biobank

Borealis of Northern Finland, Finnish Clinical Biobank Tampere, Biobank of Eastern Finland, Central Finland Biobank, Finnish Red Cross Blood Service Biobank, Terveystalo Biobank and Arctic Biobank. All Finnish biobanks are members of the BBMRI.fi infrastructure. The FINBB is the coordinator of BBMRI-ERIC operations in Finland. The Finnish biobank data can be accessed through the Fingenious® services managed by FINBB. The Finnish Biobank Cooperative (FINBB) coordinates BBMRI-ERIC operations in Finland and manages the Fingenious® services through which we accessed the data.
