# Supplementary figures and images for: ReverseGWAS identifies combined phenotypes associated with a genotype in GWA studies
Source: Bioinformatics. 2026 Feb 17;42(3):btag079. doi: 10.1093/bioinformatics/btag079 (PMC13003317; doi:10.1093/bioinformatics/btag079)

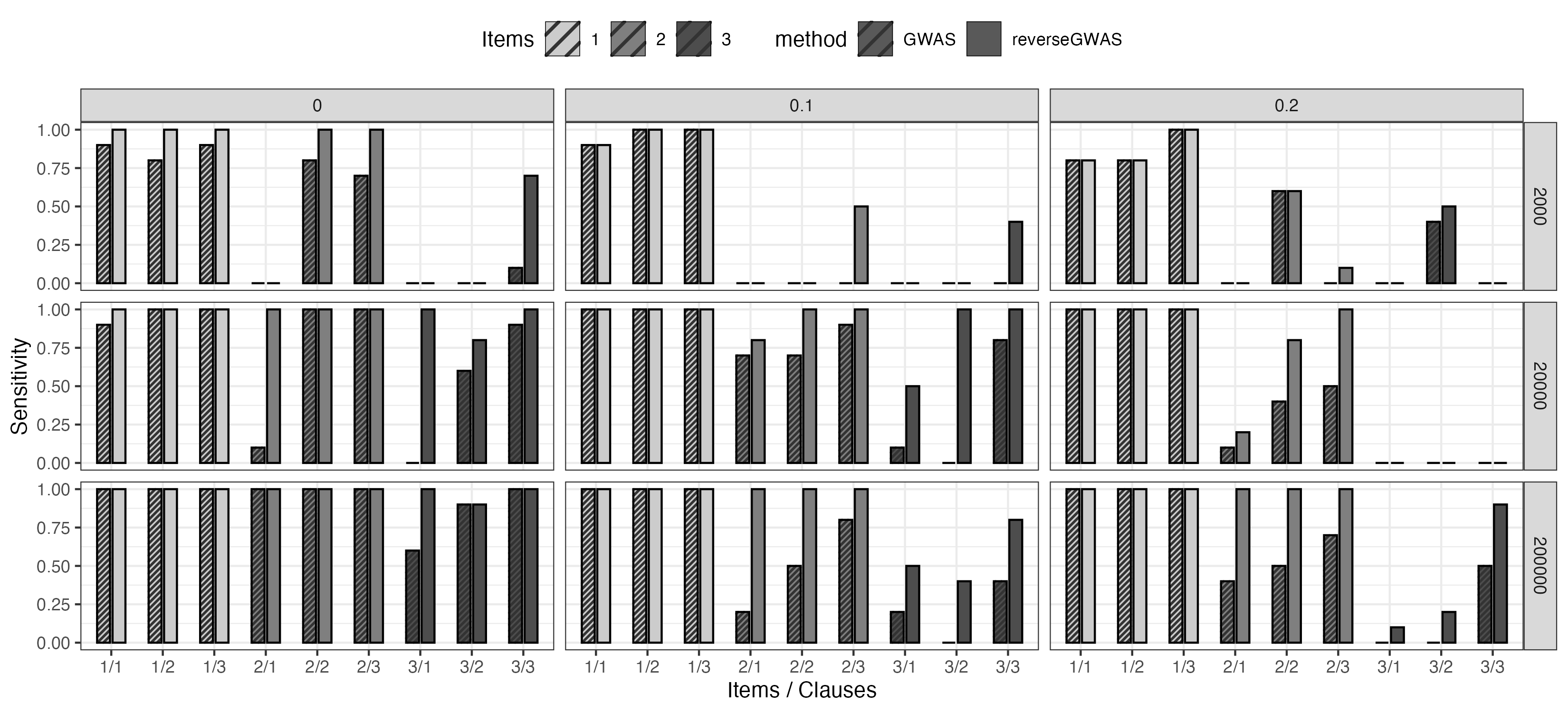

Supplement: btag079_Supplementary_Data [file btag079_supplementary_data.zip › SupplementaryFigure1.png]
